# Prognostic factors for surgical treatment of prolactin-secreting pituitary adenomas

**DOI:** 10.3389/fsurg.2024.1283179

**Published:** 2024-02-05

**Authors:** Oleksandr Voznyak, Iaroslav Zinkevych, Andrii Lytvynenko, Nazarii Hryniv, Roman Ilyuk, Nazarii Kobyliak

**Affiliations:** ^1^ Centre of Neurosurgery, Clinical Hospital “Feofaniya”, Kyiv, Ukraine; ^2^ Medical Laboratory CSD, Kyiv, Ukraine; ^3^ Endocrinology Department, Bogomolets National Medical University, Kyiv, Ukraine

**Keywords:** prolactinomas, dopamine agonists, prolactin, pituitary adenomas, transsphenoidal surgery

## Abstract

**Introduction:**

Usually, prolactinomas are treated with dopamine agonists (DA). Surgery is considered an option when the patient cannot bear or does not respond positively to DA therapy.

**Aim:**

This study aims to determine the early and late outcomes of surgery, with particular emphasis on developing prognostic factors for surgical treatment and analyzing risk factors affecting the recurrence of hyperprolactinemia and prolactinoma.

**Material and methods:**

This retrospective study was conducted at the Feofaniya Clinical Hospital of the State Administration of Affairs (Kyiv, Ukraine), evaluating 109 patients' records from 2009 to 2019. The main patients' inclusion criteria were: serum prolactin (PRL) level of more than 100 ng/ml, presence of pituitary adenoma (PA) on MRI, histologically approved PA by microscopy. According to the size of the prolactin-secreting PA (PSPAs) the selected 109 patients were divided into two groups: micro- (≤10 mm, *n* = 75) and macroadenoma group (10–40 mm, *n* = 34).

**Results:**

1 month after the operation, PRL levels decreased by 87% (*p* < 0.001), 12 months—by 93% (*p* < 0.001). After receiving surgery and DA therapy for 12 months 77.1% of patients achieved biochemical remission. Out of the total number of patients observed, 15.6% (*n* = 17) had a Knosp score greater than 3. Additionally, in the macroadenoma group, the percentage of patients with a Knosp score greater than 3 was 41,2%, which was significantly higher as compared to the microadenoma group (4%, *p* < 0.001). In patients with microadenomas a weak reverse correlation between patients' age (*r* = −0.258, *p* < 0.026) and positive with tumor size (*r* = 0.251, *p* < 0.030) was revealed. In the macroadenoma group significant association was found only between preoperative serum PRL level and tumor size (*r* = 0.412, *p* < 0.016). The preoperative PRL can be used as a diagnostic marker for lack of early biochemical remission in patients with PSPAs with diagnostic accuracy 66.9%.

**Conclusions:**

This study found that primary transsphenoidal surgery is an effective treatment in reaching PRL level control in patients with both micro- and macroprolactinomas. The correct and thorough selection of candidates for surgery is crucial to achieve postoperative serum PRL normalization in the vast majority of patients.

## Introduction

Prolactin-secreting tumors (prolactinomas) are among the most common types of pituitary tumors, accounting for about 60% of hormone-secreting tumors of the hypophysis (1–6). Overall pituitary adenomas (PA) detection frequency is 30% of clinical cases (7)*.* The prevalence and incidence of prolactin-secreting pituitary adenomas (PSPAs) is approximately 50 per 100,000 and 3–5 new cases per 100,000 per year, respectively (8). In the pediatric and adolescent age group, prolactinomas are relatively rare intracranial tumors, with a prevalence of 100 per million, representing less than 2% of all such tumors (1). Prolactinomas occur most often in women between the ages of 20 and 50, with a female-to-male ratio of approximately 10:1. In patients with multiple endocrine neoplasia type 1 (MEN-1), prolactinomas are present in around 30% of cases and tend to be more aggressive in nature compared to their sporadic counterparts. Individuals diagnosed with Carney complex or McCune-Albright syndrome may experience hyperprolactinemia caused by a pituitary tumor that arises from somatomammotropic cells. These cells secrete both growth hormone (GH) and prolactin (PRL) (2).

According to 2021 WHO Classification of Tumors of the Central Nervous System adenohypophyseal tumors were recently renamed to pituitary neuroendocrine tumors (PitNET), which are mostly benign, but may present various behaviors: invasive, “aggressive” and malignant with metastases. They are classified into seven morphofunctional types and three lineages: lactotroph, somatotroph and thyrotroph (PIT1 lineage), corticotroph (TPIT lineage) or gonadotroph (SF1 lineage), null cell or immunonegative tumor and plurihormonal tumors (9, 10).

The problem with diagnosing true prolactinomas is that hyperprolactinemia can be secondary to pituitary stalk compression by a non-secreting PA or another tumor of the sellar region (11). Also, hyperprolactinemia in both women and men can be caused by “non-pituitary” factors (12). Serum PRL levels >500 ng/ml is generally always indicative of prolactinomas (13). Macroadenomas (tumor size > 10 mm) often exhibit serum PRL higher than 250 μg/L, while microprolactinomas (tumor size < 10 mm) commonly result in hyperprolactinemia with the range between 100 and 200 μg/L (14, 15). Hyperprolactinemia of less than 100 μg/L is often related to the diagnostic uncertainty (14, 15).

To date, the standard of PSPAs treatment is the appointment of dopamine agonists (DA) (1, 16, 17), which gives an immediate result in approximately 90% of cases but provides long-term remission in no more than 30% of patients (18, 19). Therefore, patients recommended DA therapy should be warned about the high probability of lifelong drug use.

The effectiveness of radiotherapy for prolactinoma treatment is doubtful and has minimal application (1). A small proportion of patients with prolactinomas are resistant to drug therapy (3%–12%) or do not tolerate the side effects of drugs (3%–11%) (20, 21). In this group of patients with unsuccessful DA therapy, surgical treatment is recommended and postoperative permanent remission is about 72%, according to literature data (21, 22).

Some studies have shown promising results with trans-sphenoidal surgery (TSS) as the first-line prolactinoma therapy (23–25). It was demonstrated that TSS might represent a valid alternative to DA therapy, particularly in females with microadenomas, as it provides the highest chance of an immediate cure and long-term remission (26). Successful resection of PSPAs is associated with smaller sizes and lower preoperative prolactin levels (27, 28). Some patients prefer the immediate risks of surgical resection to long-term or lifelong drug therapy. It has been shown that 88% of the patients not treated by DA experienced normalizing serum PRL levels after surgery with minimal complications. It has also been established that pituitary surgery may be cost-effective compared to life-long drug therapy in patients with a life expectancy > 10 years (29, 30).

Thus, to date, there is a debate between endocrinologists and neurosurgeons regarding the expansion of traditional indications for TSS and reducing the frequency of side effects (31, 32).

This work aimed to determine the early and late outcomes of surgery, with particular emphasis on developing prognostic factors for surgical treatment and analyzing risk factors affecting the recurrence of hyperprolactinemia and prolactinoma.

## Material and methods

### Ethics statement

This single-center retrospective study was conducted at the Feofaniya Clinical Hospital of the State Administration of Affairs (Kyiv, Ukraine), evaluating 109 patients records from 2009 to 2019. The local Ethics Committee approved the research protocol and put it into practice based on the Declaration of Helsinki (1975). Informed consent was obtained from all participants included in study. One senior neurosurgeon performed whether purely microscopic transsphenoidal surgery (MTSS) or combined with endoscopic transsphenoidal surgery (ETSS) for pathologically confirmed PSPAs.

### Inclusion criteria

The main patient's inclusion criteria were: serum PRL level of more than 100 ng/ml, presence of PA on magnetic resonance imaging (MRI), histologically approved PSPAs by microscopy and, in some cases, immunohistochemically. The cut-off for PRL > 100 ng/ml was based on Endocrine Society clinical practice guideline for diagnosis and treatment of hyperprolactinemia (13). According to current guidelines microprolactinomas commonly result in hyperprolactinemia with the range between 100 and 200 ng/ml.

### Exclusion criteria

Patients with hyperprolactinemia but with a PRL level lower than 100 ng/ml, patients with pituitary apoplexy and/or nasal liquorrhea on the background of taking DA preparations were not included. The analysis did not include patients with PA who had plurihormonal activity. Also, we did not include patients who previously underwent surgeries on the pituitary gland or in the sellar region.

### Study design and outcomes assessment

According to the size of the PSPAS the selected 109 patients were divided into two groups: micro- (≤10 mm, *n* = 75) and macroadenoma group (10–40 mm, *n* = 34). Variables collected from the patient charts included age, sex, symptom duration and presence of the following symptoms: dysmenorrhea, galactorrhea, obesity, libido decrease, erectile dysfunction, hypopituitarism. The effectiveness of standard therapy by DA and doses of drugs were also analyzed.

All patients underwent a complete clinical and instrumental examination, including history taking, general clinical and neurological examination, laboratory and MRI examination.

A single MRI research associate performed all tumor volume measurements. The RECIST criteria was used to determine tumor size. The largest of 3 radiographic dimensions (anterior-posterior, transverse, and cranio-caudal) was considered representative of the tumor size. Prolactinomas were classified as microprolactinomas (diameter ≤ 10 mm) or macroprolactinomas (diameter > 10 mm). Cavernous sinus invasiveness was determined by image analysis of coronal MRI according to the Knosp criteria (33). The Knosp score determines the invasiveness of sellar masses into the cavernous sinus by depicting their relation to the intercarotid line [a theoretical line connecting both cross-sections of the internal carotid artery (ICA) on a coronal section of the cavernous sinus]; grade 0 represents a mass that does not meet or pass the intercarotid line, grades 1–3 indicate increasing levels of invasion between and past the ICA, and grade 4 represents complete encasement of the intracavernous ICA (33, 34). For statistical analysis, Knosp scores were dichotomized as ≤2 or ≥3. We recommend postoperative MRI in 3 months after surgery to control the extent of removal.

Preoperative serum PRL values preceding treatment and postoperative serum PRL values were recorded. Serum prolactin concentrations were measured with an electrochemiluminescence immunoassay (Elecsys, Roche Diagnostics). The normal PRL was considered as <20 ng/ml for males and <25 ng/ml for women. Biochemical remission was determined as normalization of serum PRL levels measured at early (1 month) and late time points (12 months) postoperatively without the need for adjuvant DA therapy. Postoperative serum PRL level lower than 10 ng/ml was used as a predictive factor for long-term surgical remission (35).

Documented postoperative complications included cerebrospinal fluid (CSF) leak required surgical repair, visual and oculomotor nerve disorders, and development of diabetes insipidus (DI). Transient DI was defined based on the full resolution of DI symptoms at follow-up, and permanent DI was defined based on persistent symptoms requiring daily desmopressin treatment. The surgical intervention was considered ineffective if the serum PRL level did not return to normal within 4 weeks after the surgery. Conversely, hyperprolactinemia recurrence after PA resection was defined as relapse of increased serum PRL level which required medical therapy, radiation therapy, or repeat surgery. Study participants had at least 12 months of follow-up as agreed by an endocrinologist.

### Surgical technique

In all cases, surgical removal of tumors was performed, and patients had undergone surgery for the first time. We performed a standard mononostril paraseptal transsphenoidal approach to sella turcica. During recent years microsurgical technique was gradually replaced by endoscopic surgery, but we still characterize our technic as “hybrid” because we still use the operative microscope for at least transsphenoidal approach. We performed a wide horizontal opening of the sella turcica between the cavernous sinuses and a vertical one from the planum sphenoidale to the clivus. To gain full visibility of the anterior surface of the pituitary gland, we used an H-shaped incision in the dura mater. We always detected the PA laterally and removed it using microsurgical dissectors, cup curettes, ring curettes, and an aspirator. Following complete hemostasis, we conducted a Valsalva test to ensure no CSF presence in the operating field.

In cases of CSF leakage, we used fat tissue harvested from the hypogastric area for fistula hermetization. We performed sella turcica floor reconstruction by bone plate obtained during surgical approach in all cases. We believe that the “closed” sella turcica provides optimal conditions for the functioning of the pituitary gland in the postoperative period.

### Pathohistological diagnosis of PSPAs

All tumors were histologically verified. All specimens were cut and stained with hematoxylin and eosin (H&E) to classify PA. In addition, in some cases, specific pituitary hormones such as growth hormone (GH), PRL, and ACTH were identified using immunohistochemical (IHC) staining with monoclonal antibodies. Ki-67 expression was also evaluated on a portion of the specimens. Experienced pathologists verified histopathological diagnosis in line with the 2017 World Health Organization (WHO) classification (36).

### Statistical analysis

Statistical analysis was done using a standard software SPSS version 20.0 (SPSS, Inc., Chicago, Illinois) and GraphPad Prism, version 6.0 (GraphPad Software, Inc., La Jolla, CA, USA). Data distribution was analyzed using the Kolmogorov-Smirnov normality test. All continuous values are expressed as mean ± SD and categorical variables are presented as %. For comparison 3 continuous variables with parametric distribution were then analyzed using Analysis of Variance (ANOVA) and if the results were significant, a Bonferroni Post Hoc test was performed. The independent samples t-test was used to compare differences between micro- and macroadenoma groups. Data with nonparametric distribution was analyzed using Kruskall-Wallis test. For comparisons of categorical variables we conducted a *χ*^2^ test. Association between preoperative PRL levels and tumor size was assessed with univariate Pearson's correlation analysis.

To assess the diagnostic accuracy of preoperative PRL for predicting absence of remission we used receiver operating characteristic (ROC) curves. The ROC curve is a plot of sensitivity (Se) vs. 1-specificity (Sp) for all possible cut-off values. The most commonly used index of accuracy is area under the ROC curve (AUROC). AUROC values close to 1.0 indicated high diagnostic accuracy. Optimal cut-off values were chosen to maximize the sum of sensitivity and specificity, and positive (PPV) and negative predictive values (NPV) were computed for these cut-off values (37).

## Results

During the analyzed period of 10 years, 623 patients with PA were operated on in our clinic, 369 of them had hyperprolactinemia before surgery, but only 109 patients met the criteria for inclusion in the study. Table 1 shows the baseline characteristics and symptoms in examined patients operated on PSPAS. The mean age of the 109 patients involved in the study was 34.3 ± 11.17 years. Common symptom presentations included: symptoms duration before surgery (15.44 ± 6.59 months), erectile dysfunction (43.1%), dysmenorrhea (53.2%), galactorrhea (21.1%), obesity (33.9%), libido decrease (64.2%), hypopituitarism (13.8%), non-specific (25.7%), asymptomatic (2.8%). Pretreatment with DA was received by 66.1% of patients (cabergoline 54.1% and bromocriptine 11%). Non-efficacy of DA therapy was documented in 53.2% of operated patients (Table 1).

**Table 1:** The baseline characteristics and symptoms in examined patients operated on PSPAs.

Parameter	All patients (*n* = 109)	Micro (*n* = 75)	Macro (*n* = 34)	*p*1	*p*2
Age, years	34.3 ± 11.17	33.59 ± 10.98	35.88 ± 11.59	0.323	0.611
Symptom duration, month	15.44 ± 6.59	15.4 ± 6.95	15.53 ± 5.82	0.925	0.996
Erectile dysfunction, % (*n*)	43.1 (47)	46.7 (35)	35.3 (12)	0.267	0.540
Dysmenorrhea, % (*n*)	53.2 (58)	53.3 (40)	52.9 (18)	0.970	0.999
Galactorrhea, % (*n*)	21.1 (23)	17.3% (13)	29.4 (10)	0.152	0.359
Obesity, % (*n*)	33.9 (37)	33.3 (25)	35.3 (12)	0.841	0.980
Libido decrease, % (*n*)	64.2 (70)	66.7 (50)	58.8 (20)	0.429	0.731
Hypopituitarism, % (*n*)	13.8 (15)	12.0 (9)	17.6 (6)	0.563	0.730
Non-specific, % (*n*)	25.7 (28)	25.3 (19)	26.5 (9)	0.900	0.992
Asymptomatic, % (*n*)	2.8 (3)	1.3 (1)	5.9 (2)	0.179	0.405
Pretreatment with DA, % (*n*)	66.1 (72)	72.0 (54)	50.0 (17)	0.026	0.083
Cabergoline, % (*n*)	54.1 (59)	60.0 (45)	41.2 (14)	0.068	0.188
Cabergoline dosage, mg	1.97 ± 0.36	2.0 ± 0.37	1.89 ± 0.30	0.336	0.626
Bromocriptine, % (*n*)	11.0 (12)	12.0 (9)	8.8 (3)	0.624	0.887
Indication, % (*n*)				0.102	0.400
DA non-efficacy	53.2 (58)	58.7 (44)	41.2 (14)		
Patient selection	36.7 (40)	29.3 (22)	52.9 (18)		
Repeated	2.8 (3)	2.7 (2)	2.9 (1)		
Intolerance	7.3 (8)	9.3 (7)	2.9 (1)		

*p*1, difference between micro- and macroadenoma groups; *p*2, differences between all 3 variables. Significance was stated at *p* < 0,05.

All patients were divided into two groups according to the size of the PSPASs: micro- (*n* = 75) and macroadenoma groups (*n* = 34). The enrolled patients' age, baseline characteristics and symptoms did not significantly differ between groups, except for one indicator, pretreatment with DA.

**Figure 1:**
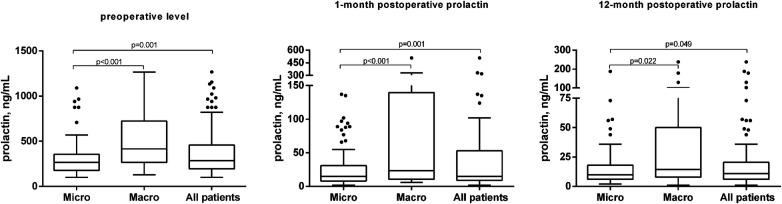
Dynamic of PLR levels before the operation, one month and twelve months after.

At the time of diagnosis, the mean PRL level was 377.55 ± 265.42 ng/ml and tumor size—10.10 ± 7.0 mm. The size of tumors in the microadenoma group was 6.4 ± 1.91 mm, while it was 18.26 ± 7.23 mm in the macroadenoma group (*p* < 0.001) (Table 2). The preoperative PRL level in the microadenoma group was 312.29 ± 211.42 ng/ml, significantly higher than the normal hormone level in the blood. The preoperative PRL level in the macroadenoma group was 521.5 ± 316.8, which was 67% higher (*p* < 0.001) as compared to patients with microadenomas (Table 2, Figure 1A). One month after the operation, PRL levels decreased by 87% (*p* < 0.001) to 48.75 ± 78.9 ng/ml. Twelve months after the procedure, PRL levels decreased by 93% (*p* < 0.001) to 25.33 ± 39.02. A similar trend was observed when comparing prolactin levels in two groups (Table 2, Figures 1B,C). A greater number of patients from microadenoma group achieve early (69.3 vs. 50.0%, *p* = 0.052) and late (80. vs. 70.6%, *p* = 0.279) biochemical remission, but changes were insignificant as compared to macroadenoma group. After one month of surgery, 63.3% of patients experienced biochemical remission (with PRL levels less than 10%–35.7%). After 12 months of surgery and DA therapy, 77.1% of patients achieved biochemical remission (with PRL levels less than 10%–46.8%) (Table 2).

**Table 2:** Preoperative characteristics and biochemical remission in patients who underwent surgery for PSPAs.

Parameter	All patients (*n* = 109)	Micro (*n* = 75)	Macro (*n* = 34)	*p*1	*p*2
Tumor size, mm	10.10 ± 7.0^a^	6.4 ± 1.91^b^	18.26 ± 7.23^c^	<0.001	<0.001
Preoperative PRL, ng/ml	377.55 ± 265.42^a^	312.29 ± 211.42^a^	521.5 ± 316.8^b^	<0.001	0.001
1-month postoperative PRL, ng/ml	48.75 ± 78.9^a^	29.85 ± 35.58^a^	90.44 ± 122.2^b^	<0.001	0.001
12-month postoperative PRL, ng/ml	25.33 ± 39.02^a,b^	19.61 ± 28.06^a^	37.97 ± 54.57^b^	0.022	0.049
Knosp grade, % (*n*)				<0.001	<0.001
0	37.6 (41)	45.3 (34)	20.6 (7)		
1	22.0 (24)	30.7 (23)	2.9 (1)		
2	24.8 (27)	20.0 (15)	35.3 (12)		
3	10.1 (11)	2.7 (2)	26.5 (9)		
4	5.5 (6)	1.3 (1)	14.7 (5)		
Knosp ≤ 2, % (*n*)	84.4 (92)	96 (72)	58.8 (20)	<0.001	<0.001
Knosp > 3, % (*n*)	15.6 (17)	4 (3)	41.2 (14)	<0.001	<0.001
Cyst structure, % (*n*)	42.2 (46)	41.3 (31)	44.1 (15)	0.785	0.964
1-month PRL < 10, % (*n*)	35.7 (39)	40.0 (30)	26.5 (9)	0.172	0.388
Biochemical remission after 1-month, % (*n*)	63.3 (69)	69.3 (52)	50.0 (17)	0.052	0.152
12-month PRL < 10, % (*n*)	46.8 (51)	52.0 (39)	35.3 (12)	0.105	0.267
Biochemical remission after 12-month with DA therapy, % (*n*)	77.1 (84)	80.0 (60)	70.6 (24)	0.279	0.556

*p*1, difference between micro- and macroadenoma groups; *p*2, differences between all 3 variables.

^a,b,c^ Values on the same row with different superscript letters show significant differences in *p* < 0.05.

**Figure 2:**
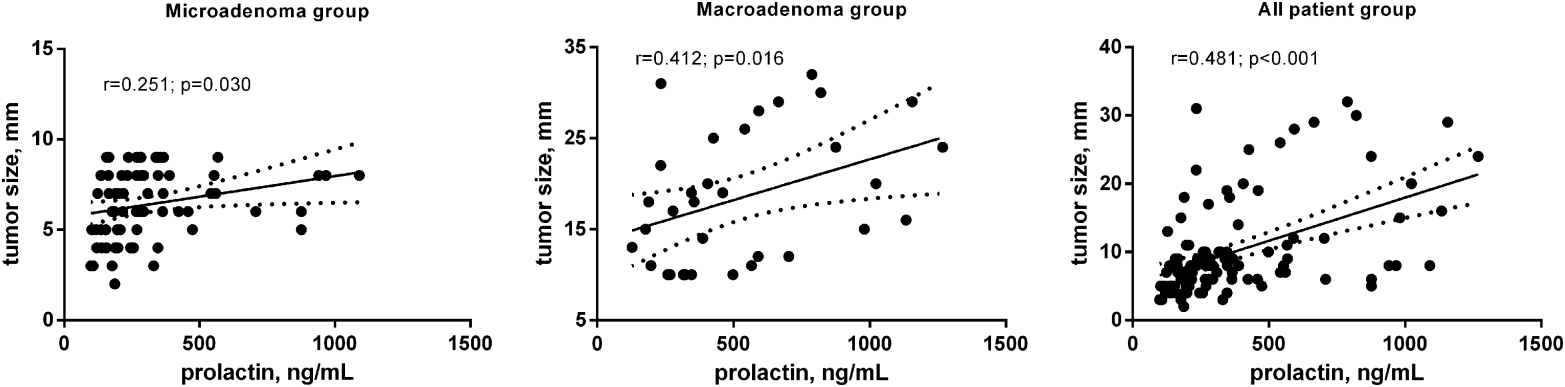
Correlation between preoperative PLR level (ng/ml) and tumor size (mm) in patients after transsphenoidal resection for PSPAs.

It has analyzed the correlation between preoperative PLR level and other parameters: age, tumor size and cavernous sinus invasion. In patients with microadenomas a weak reverse correlation between patients' age (*r* = −0.258, *p* < 0.026) and positive with tumor size (*r* = 0.251, *p* < 0.030) was revealed (Table 3, Figure 2A). In the macroadenoma group significant association was found only between preoperative serum PLR level and tumor size (*r* = 0.412, *p* < 0.016) (Table 3, Figure 2B). The power of correlation was more pronounced in patients with macroadenomas.

**Table 3:** Correlation analysis between preoperative PRL level and other parameters.

Parameter	All patients (*n* = 109)	Micro (*n* = 75)	Macro (*n* = 34)
Age, years	−0.128 (0.184)	−0.258 (0.026)^a^	−0.067 (0.707)
Tumor size, mm	0.481 (<0.001)	0.251 (0.030)^a^	0.412 (0.016)^a^
Knosp grade	0.251 (0/008)	0.117 (0.317)	−0.013 (0.943)

The data presented as r (*p*).

^a^ Significant correlation.

Based on Knosp classification, in 62% (*n* = 68) cases cavernous sinus invasion was presented. The Knosp grade distribution was as follows: grade 0 in 37.6% (*n* = 41), grade 1 in 22.0% (*n* = 24), grade 2 in 24.8% (*n* = 27), grade 3 in 10.1% (*n* = 11), and grade 4 in 5.5% (*n* = 6) (Table 2). Out of the total number of patients observed, 15.6% (*n* = 17) had a Knosp score greater than 3. Additionally, in the macroadenoma group, the percentage of patients with a Knosp score greater than 3 was 41,2%, which was significantly higher as compared to the microadenoma group (4%, *p* < 0.001) (Table 2). Also, we did not find significant correlation between preoperative PRL and Knosp grade (Table 3).

Regarding postoperative complications, patients are observed on average 15.2 ± 7.33 months. Among the postoperative complications, the most frequently reported were CSF leakage during the operation in 10.1% of patients and after the operation in 1.8% (Table 4). These violations were observed in both groups and did not differ statistically significantly. Also, no statistically significant differences were observed between the two groups in the residual tumor after the operation (5.5% of all patients), permanent diabetes insipidus (2.8% of all patients) and transient diabetes insipidus (7.3% of all patients). It appears that some postoperative complications were found just in the group of patients with macroadenomas, and their prevalence was significantly higher as compared to patients with microadenomas: visual disorders accounted for about 1.8% of the total patient population (*p* < 0.034), hypopituitarism after the operation—3.7% (*p* < 0.002). Additionally, there was one patient who also experienced oculomotor disorders—0.9% of all operated subjects (Table 4).

**Table 4:** The postoperative complication in examined patients operated on PSPAs.

Parameter	All patients (*n* = 109)	Micro (*n* = 75)	Macro (*n* = 34)	*p*1	*p*2
Follow up, years	15.2 ± 7.33	14.87 ± 6.95	15.94 ± 8.17	0.481	0.779
Visual disorders, % (*n*)	1.8 (2)	–	5.9 (2)	0.034	0.106
Oculomotor disorders, % (*n*)	0.9 (1)	–	2.9 (1)	0.136	0.329
Residual tumor after operation, % (*n*)	5.5 (6)	2.7 (2)	11.8 (4)	0.054	0.155
Hypopituitarism after operation, % (*n*)	3.7 (4)	–	11.8 (4)	0.002	0.010
CFS leak intra-operation, % (*n*)	10.1 (11)	6.7 (5)	17.6 (6)	0.078	0.211
CFS leak after operation, % (*n*)	1.8 (2)	1.3 (1)	2.9 (1)	0.562	0.845
Permanent diabetes insipidus, % (*n*)	2.8 (3)	1.3 (1)	5.9 (2)	0.179	0.405
Transient diabetes insipidus, % (*n*)	7.3 (8)	5.3 (4)	11.8 (4)	0.233	0.491

*p*1, difference between micro- and macroadenoma groups; *p*2, differences between all 3 variables. Significance was stated at *p* < 0,05.

**Figure 3:**
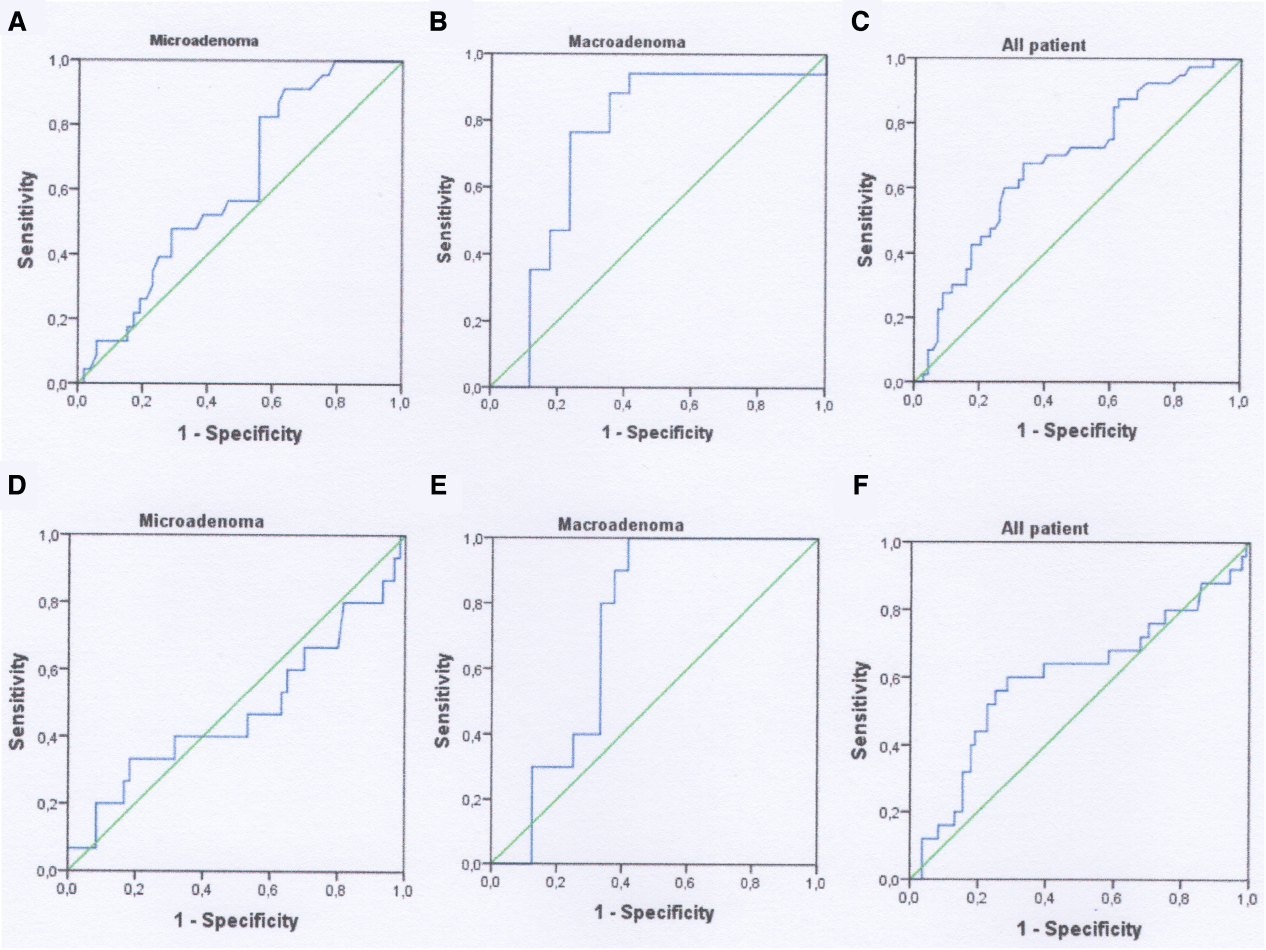
ROC-curves using as a predictor for lack of remission preoperative PRL. (**A–C**) 1 month analysis; (**D–F**) 12-month analysis.

According to ROC-analysis, preoperative PRL may be used as a diagnostic marker for predicting lack of biochemical remission only after 1-month in all patient's populations. The level of PRL greater than 299.5 ng/ml with sensitivity—67.5%, specificity—66.6%, PPV—54.0% and NPV—77.9% can predict absence of early remission. The AUROC for the model was 0.680 (95% CI 0.577–0.783, *p* = 0.015) (Figure 3, Table 5). Cavernous sinus invasion maybe possible confounding factor that can impact the importance of the level of preoperative PRL as diagnostic marker. We additionally tested this hypothesis and excluded patient with Knosp 3–4 from ROC-analysis. After the standardization for this confounding factor the accuracy of model didn't change in all patients group. The AUROC for the model after standardization was 0.670 (95% CI 0.560–0.781, *p* = 0.006).

**Table 5:** ROC analysis for predicting the absence of biochemical remission when using preoperative PRL level as a diagnostic marker.

Parameter	All patients (*n* = 109)	Micro (*n* = 75)	Macro (*n* = 34)
Analysis after 1 month			
Cut-off value	>299.5	>200	>345
Sensitivity, %	67.5	44.2	70.5
Specificity, %	66.6	82.6	88.2
NPV, %	77.9	85.1	85.7
PPV, %	54.0	39.5	75.0
Diagnostic accuracy, %	66.9	56.0	79.4
AUROC	0.680	0.616	0.744
95% CI	0.577–0.783	0.487–0.745	0.561–0.927
P (AUROC)	0.002	0.112	0.015
Analysis after 12 months			
Cut-off value	>351	>210	>396
Sensitivity, %	60.0	53.3	90.0
Specificity, %	71.4	36.6	62.5
NPV, %	85.7	75.8	93.7
PPV, %	38.4	17.3	50.0
Diagnostic accuracy, %	68.8	40.0	70.5
AUROC	0.599	0.477	0.725
95% CI	0.459–0.738	0.293–0.660	0.558–0.892
P (AUROC)	0.136	0.781	0.041

NPV, negative predictive value; PPV, positive predictive value; AUROC, area under ROC-curve, 95% CI—95% confidence interval for AUROC.

Preoperative PRL can significantly predict lack of biochemical remission in patients with macroadenomas without taking account cavernous sinus invasion. In patients with absence of biochemical remission after 1 month the quality of this diagnostic model was moderate since AUROC for PRL was 0.744 (95% CI 0.561–0.927, *p* = 0.015) (Figure 3, Table 5). The cut-off value of preoperative PRL for predicting lack of remission was >345 ng/ml. Sensitivity, specificity, PPV and NPV were 70.5%, 88.2%, 75.0%, 85.7% respectively. For 12-month lack remission AUROC constituted 0.725 (95% CI 0.558–0.892, *p* = 0.041), with cut-off for preoperative PRL > 396 ng/ml. The overall diagnostic accuracy of the model was 70.5%, that was lower as compared to 1-month data (Figure 3, Table 5). However, after the standardization for Knosp 3–4 grade, the overall quality of models for both early and late lack of remission were insignificant: AUROC for 1-month absence of remission—0.670 (95% CI 0.384–0.597, *p* = 0.219) and for 12-month—0.655 (95% CI 0.416–0.894, *p* = 0.284) respectively.

## Discussion

Traditionally, the preferred treatment for PSPAs is medical therapy involving DA. The typical reasons for surgery are when patients cannot tolerate or do not respond well to DA therapy. In our study, 66% of patients took DA before surgery. In the macroadenoma group, significantly (*p* < 0.026), fewer patients were on therapy with DA as compared to the microadenoma group. This fact could be explained by pituitary macroadenomas typically present with mass effects — meaning their large size can apply pressure to or damage nearby tissues, causing compressive symptoms and patients do not have time for classical therapy (38).

Surgical treatment becomes the second-line therapy option when medical management fails or cannot be accepted. For successful results, patient selection is crucial, as factors such as large tumor size and cavernous sinus invasion can harm prognosis (39). Moreover, endoscopic endonasal transtuberculum/transplanum approach (EEA-TTP) for giant PAs is a valid option and seems to provide better outcomes, lower rate of complications and higher extent of resection compared to one- or multi-stage microscopic, non-extended endoscopic transsphenoidal, and transcranial resections (40).

Kreutzer J. et al. looked back at past surgical cases to see if there were any changes in the reasons for surgery. It was found that fewer patients were being operated on for traditional reasons, but there was a significant increase in patients choosing surgery as their primary treatment option. 53.2% of patients, including those with giant PSPAs, achieved initial remission (27).

In our study, the success rate for remission after one year of surgical treatment for prolactinomas was high, with a rate of 77.1%. In microadenomas, the success rate is even higher, at 80.0%. One of the most reported postoperative complications is leakage of CSF during surgery. Biochemical remission was achieved one month after surgery in 63.3% of patients. After receiving surgery and DA therapy for 12 months 77.1% of patients achieved biochemical remission.

It was discovered a connection between the tumor size and the preoperative PLR level. Patients who did not experience remission had a higher increase in PRL levels for each rise in preoperative tumor volume than those who achieved remission (41). This suggests that there may be a distinct tumor composition in these cases. In our investigation, a correlation was discovered in the microadenoma group between preoperative PLR levels and age, also with tumor size. In the macroadenoma group, the correlation was only between serum PLR levels and tumor size. The preoperative PRL can be used as a predictor of both early and long-term biochemical remission only in the macroadenomas group with diagnostic accuracy greater than 70%.

Surgeons and therapists are currently discussing an essential question regarding whether surgery for PSPAs will be a more cost-effective option than lifelong medical management (39). Studies have shown that patients with intrasellar microadenomas that do not involve the cavernous sinus and have preoperative PRL levels below 200 ng/ml can have outcomes that are as good as or better than those who receive medical management (39, 42, 43). TSS can result in a surgical cure rate of up to 90% in many cases (39, 44, 45).

For example, the costs of medical procedures showed that a single patient undergoing transsphenoidal PSPAs surgery could incur about $10,000 in surgical and perioperative costs. Additionally, yearly DAs medication costs approximately $3300, and the cost of medical management would exceed that of surgery within four years. Furthermore, medically managed patients may require serial imaging to monitor the size of the adenoma. The authors found that TSS is a more cost-effective option for experienced pituitary surgeons, with an expected near 0% mortality rate and minimal morbidity (46, 47).

Regarding the quality of life for patients with PSPAs, there have been reports of it being negatively impacted after undergoing medical management or TSS (39, 48–50). Studies have demonstrated that surgically reducing the size of prolactinomas can enable patients to achieve normal PRL levels post-surgery with lower medication (51). This can lead to more manageable side effects for patients. Certain patient groups may probably benefit from surgical resection, particularly young patients with intrasellar adenomas without suprasellar or cavernous sinus invasion, due to the accessibility of the lesion and high cure rates. We hope that our findings can be used to identify PSPAs for which primary TSS may achieve biochemical remission as an alternative to DAs therapy.

The retrospective design with relatively short follow up time is the main limitation of current study. Our study was also performed at a single surgery center specialized on management of patients with PA. Thus, our patient selection and postoperative complications may not be generalizable to institutions without a similar case volume. Additionally, we included in the study 31 patient with preoperative PRL level between 100 and 200 ng/ml. Among several studies such PRL level can be recognized as “grey zone” and belongs to possible pitfalls especially differentiating microprolactinomas with non-functional PA. Finally, although we determined the optimal preoperative PRL cut-off level for prediction of postoperative lack of remission, it must be emphasized that these values are not absolute predictors. False positives and negatives are always possible when using a single test to predict outcomes.

## Conclusion

This study found that primary transsphenoidal surgery is an effective treatment in reaching PRL level control in patients with both micro- and macroprolactinomas. The correct and thorough selection of candidates for surgery is crucial to achieve postoperative serum PRL normalization in the vast majority of patients. The preoperative PRL can be used as a diagnostic marker for lack of early biochemical remission in patients with PSPAs with diagnostic accuracy 66.9%.

## Data Availability

The raw data supporting the conclusions of this article will be made available by the authors, without undue reservation.

## References

[1] Chanson P Maiter D. The epidemiology, diagnosis and treatment of prolactinomas: the old and the new. Best Pract Res Clin Endocrinol Metab. (2019) 33:101290. 10.1016/j.beem.2019.101290 31326373

[2] Ciccarelli A Daly AF Beckers A. The epidemiology of prolactinomas. Pituitary. (2005) 8:3–6. 10.1007/s11102-005-5079-0 16411062

[3] Zielinski G Ozdarski M Maksymowicz M Szamotulska K Witek P. Prolactinomas: prognostic factors of early remission after transsphenoidal surgery. Front Endocrinol. (2020) 11:439. 10.3389/fendo.2020.00439 PMC7358351 32733387

[4] Daly AF Tichomirowa MA Beckers A. The epidemiology and genetics of pituitary adenomas. Best Pract Res Clin Endocrinol Metab. (2009) 23:543–54. 10.1016/j.beem.2009.05.008 19945022

[5] Molitch ME. Pituitary incidentalomas. Best Pract Res Clin Endocrinol Metab. (2009) 23:667–75. 10.1016/j.beem.2009.05.001 19945030

[6] Gillam MP Molitch ME Lombardi G Colao A. Advances in the treatment of prolactinomas. Endocr Rev. (2006) 27:485–534. 10.1210/er.2005-9998 16705142

[7] Аденома гіпофіза: симптоми, діагностика та лікування пухлин у Харкові, Україні.

[8] Melmed S. Pituitary-tumor endocrinopathies. N Engl J Med. (2020) 382:937–50. 10.1056/NEJMra1810772 32130815

[9] Trouillas J Jaffrain-Rea ML Vasiljevic A Raverot G Roncaroli F Villa CC. How to classify the pituitary neuroendocrine tumors (PitNET)s in 2020. Cancers. (2020) 12:514. 10.3390/CANCERS12020514 32098443 PMC7072139

[10] Louis DN Perry A Wesseling P Brat DJ Cree IA Figarella-Branger D The 2021 WHO classification of tumors of the central nervous system: a summary. Neuro Oncol. (2021) 23:1231–51. 10.1093/NEUONC/NOAB106 34185076 PMC8328013

[11] Yatavelli RKR Bhusal K. Prolactinoma. In: StatPearls. Treasure Island: StatPearls Publishing (2023). 29083585

[12] Nishioka H Haraoka J Akada K Azuma S. Gender-related differences in prolactin secretion in pituitary prolactinomas. Neuroradiology. (2002) 44:407–10. 10.1007/s00234-002-0774-2 12012125

[13] Melmed S Casanueva FF Hoffman AR Kleinberg DL Montori VM Schlechte JA Diagnosis and treatment of hyperprolactinemia: an endocrine society clinical practice guideline. J Clin Endocrinol Metab. (2011) 96:273–88. 10.1210/JC.2010-1692 21296991

[14] Vilar L Vilar CF Lyra R Da Conceição Freitas M. Pitfalls in the diagnostic evaluation of hyperprolactinemia. Neuroendocrinology. (2019) 109:7–19. 10.1159/000499694 30889571

[15] Kim JH Hur KY Hong SD Choi JW Seol HJ Nam DH Serum prolactin level to tumor size ratio as a potential parameter for preoperative differentiation of prolactinomas from hyperprolactinemia-causing non-functional pituitary adenomas. World Neurosurg. (2022) 159:e488–96. 10.1016/J.WNEU.2021.12.074 34958988

[16] Delgrange E Sassolas G Perrin G Jan M Trouillas J. Clinical and histological correlations in prolactinomas, with special reference to bromocriptine resistance. Acta Neurochir. (2005) 147:751–7. 10.1007/s00701-005-0498-2 15971099

[17] Castinetti F Albarel F Amodru V Cuny T Dufour H Graillon T The risks of medical treatment of prolactinoma. Ann Endocrinol. (2021) 82:15–9. 10.1016/j.ando.2020.12.008 33373604

[18] Lu J Cai L Wu Z Lin W Xu J Zhu Z Surgery and medical treatment in microprolactinoma: a systematic review and meta-analysis. Int J Endocrinol. (2021) 2021:1–11. 10.1155/2021/9930059 PMC8423556 34504526

[19] Inder WJ Jang C. Treatment of prolactinoma. Medicina. (2022) 58:1095. 10.3390/medicina58081095 36013562 PMC9413135

[20] Webster J Piscitelli G Polli A Ferrari CI Ismail I Scanlon MF. A comparison of cabergoline and bromocriptine in the treatment of hyperprolactinemic amenorrhea. N Engl J Med. (1994) 331:904–9. 10.1056/NEJM199410063311403 7915824

[21] Fukuhara N Nishiyama M Iwasaki Y. Update in pathogenesis, diagnosis, and therapy of prolactinoma. Cancers. (2022) 14:3604. 10.3390/cancers14153604 35892862 PMC9331865

[22] Haider SA Levy S Rock JP Craig JR. Prolactinoma. Otolaryngol Clin North Am. (2022) 55:305–14. 10.1016/j.otc.2021.12.005 35256169

[23] Akin S Isikay I Soylemezoglu F Yucel T Gurlek A Berker M. Reasons and results of endoscopic surgery for prolactinomas: 142 surgical cases. Acta Neurochir. (2016) 158:933–42. 10.1007/s00701-016-2762-z 26970763

[24] Song Y-J Chen M-T Lian W Xing B Yao Y Feng M Surgical treatment for male prolactinoma. Medicine. (2017) 96:e5833. 10.1097/MD.0000000000005833 28079813 PMC5266175

[25] Zamanipoor Najafabadi AH Zandbergen IM de Vries F Broersen LHA van den Akker-van Marle ME Pereira AM Surgery as a viable alternative first-line treatment for prolactinoma patients. A systematic review and meta-analysis. J Clin Endocrinol Metab. (2020) 105:e32–41. 10.1210/clinem/dgz144 31665485 PMC7112976

[26] Mattogno PP D’Alessandris QG Chiloiro S Bianchi A Giampietro A Pontecorvi A Reappraising the role of trans-sphenoidal surgery in prolactin-secreting pituitary tumors. Cancers. (2021) 13:3252. 10.3390/cancers13133252 34209686 PMC8269319

[27] Kreutzer J Buslei R Wallaschofski H Hofmann B Nimsky C Fahlbusch R Operative treatment of prolactinomas: indications and results in a current consecutive series of 212 patients. Eur J Endocrinol. (2008) 158:11–8. 10.1530/EJE-07-0248 18166812

[28] Tyrrell JB Lamborn KR Hannegan LT Applebury CB Wilson CB. Transsphenoidal microsurgical therapy of prolactinomas: initial outcomes and long-term results. Neurosurgery. (1999) 44:254–61. 10.1097/00006123-199902000-00006 9932878

[29] Jethwa PR Patel TD Hajart AF Eloy JA Couldwell WT Liu JK. Cost-effectiveness analysis of microscopic and endoscopic transsphenoidal surgery versus medical therapy in the management of microprolactinoma in the United States. World Neurosurg. (2016) 87:65–76. 10.1016/j.wneu.2015.10.090 26548828

[30] Zygourakis CC Imber BS Chen R Han SJ Blevins L Molinaro A Cost-effectiveness analysis of surgical versus medical treatment of prolactinomas. J Neurol Surg B Skull Base. (2017) 78:125–31. 10.1055/s-0036-1592193 28321375 PMC5357228

[31] Behan LA Moyles P Cuesta M Rogers B Crowley RK Ryan J The incidence of anterior pituitary hormone deficiencies in patients with microprolactinoma and idiopathic hyperprolactinaemia. Clin Endocrinol. (2017) 87:257–63. 10.1111/cen.13355 28425105

[32] Micko A Vila G Höftberger R Knosp E Wolfsberger S. Endoscopic transsphenoidal surgery of microprolactinomas: a reappraisal of cure rate based on radiological criteria. Neurosurgery. (2019) 85:508–15. 10.1093/neuros/nyy385 30169711

[33] Knosp E Steiner E Kitz K Matula C. Pituitary adenomas with invasion of the cavernous sinus space: a magnetic resonance imaging classification compared with surgical findings. Neurosurgery. (1993) 33:610–8. 10.1227/00006123-199310000-00008 8232800

[34] Abou-Al-Shaar H Mallela AN Patel A Shariff RK Shin SS Choi PA The role of endoscopic endonasal surgery in the management of prolactinomas based on their invasiveness into the cavernous sinus. Pituitary. (2022) 25:508–19. 10.1007/S11102-022-01221-3 35467273

[35] Amar AP Couldwell WT Chen JCT Weiss MH. Predictive value of serum prolactin levels measured immediately after transsphenoidal surgery. J Neurosurg. (2002) 97:307–14. 10.3171/JNS.2002.97.2.0307 12186458

[36] Lopes MBS. The 2017 world health organization classification of tumors of the pituitary gland: a summary. Acta Neuropathol. (2017) 134:521–35. 10.1007/s00401-017-1769-8 28821944

[37] Mykhalchyshyn G Kobyliak N Bodnar P. Diagnostic accuracy of acyl-ghrelin and it association with non-alcoholic fatty liver disease in type 2 diabetic patients. J Diabetes Metab Disord. (2015) 14:44. 10.1186/s40200-015-0170-1 25995986 PMC4438435

[38] Irfan H Shafiq W Siddiqi AI Ashfaq S Attaullah S Munir Alvi A Prolactinoma: clinical characteristics, management and outcome. Cureus. (2022) 14:e29822. 10.7759/cureus.29822 36337795 PMC9626374

[39] Kim W Chivukula S Heaney A Wang M Bergsneider M. Transsphenoidal Surgery for Prolactinomas. Cham: Springer International Publishing (2017). p. 457–69. 10.1007/978-3-319-56691-7_26

[40] Chibbaro S Signorelli F Milani D Cebula H Scibilia A Bozzi MT Primary endoscopic endonasal management of giant pituitary adenomas: outcome and pitfalls from a large prospective multicenter experience. Cancers. (2021) 13:3603. 10.3390/CANCERS13143603 34298816 PMC8304085

[41] Osorio RC Pereira MP Oh T Joshi RS Haddad AF Pereira KM Correlation between tumor volume and serum prolactin and its effect on surgical outcomes in a cohort of 219 prolactinoma patients. J Neurosurg. (2022) 138:1669–79. 10.3171/2022.8.JNS221890 36242577

[42] Losa M Mortini P Barzaghi R Gioia L Giovanelli M. Surgical treatment of prolactin-secreting pituitary adenomas: early results and long-term outcome. J Clin Endocrinol Metab. (2002) 87:3180–6. 10.1210/jcem.87.7.8645 12107221

[43] Thompson C Suh J Liu Y Bergsneider M Wang M. Modifications to the endoscopic approach for anterior skull base lesions improve postoperative sinonasal symptoms. J Neurol Surg Part B Skull Base. (2013) 75:065–72. 10.1055/s-0033-1356492 PMC3912144 24498592

[44] Qu X Wang M Wang G Han T Mou C Han L Surgical outcomes and prognostic factors of transsphenoidal surgery for prolactinoma in men: a single-center experience with 87 consecutive cases. Eur J Endocrinol. (2011) 164:499–504. 10.1530/EJE-10-0961 21252173

[45] Babey M Sahli R Vajtai I Andres RH Seiler RW. Pituitary surgery for small prolactinomas as an alternative to treatment with dopamine agonists. Pituitary. (2011) 14:222–30. 10.1007/s11102-010-0283-y 21170594 PMC3146980

[46] Couldwell WT Weiss MH. Medical and surgical management of microprolactinoma. Pituitary. (2004) 7:31–2. 10.1023/B:PITU.0000044631.89535.48 15638295

[47] Bloomgarden E Molitch ME. Surgical treatment of prolactinomas: cons. Endocrine. (2014) 47:730–3. 10.1007/s12020-014-0369-9 25112227

[48] Cesar de Oliveira Naliato E Dutra Violante AH Caldas D Lamounier Filho A Rezende Loureiro C Fontes R Quality of life in women with microprolactinoma treated with dopamine agonists. Pituitary. (2008) 11:247–54. 10.1007/s11102-008-0091-9 18270842

[49] Raappana A Pirilä T Ebeling T Salmela P Sintonen H Koivukangas J. Long-term health-related quality of life of surgically treated pituitary adenoma patients: a descriptive study. ISRN Endocrinol. (2012) 2012:1–8. 10.5402/2012/675310 PMC3549391 23346413

[50] Ritvonen E Karppinen A Sintonen H Vehkavaara S Kivipelto L Roine RP Normal long-term health-related quality of life can be achieved in patients with functional pituitary adenomas having surgery as primary treatment. Clin Endocrinol. (2015) 82:412–21. 10.1111/cen.12550 25039500

[51] Primeau V Raftopoulos C Maiter D. Outcomes of transsphenoidal surgery in prolactinomas: improvement of hormonal control in dopamine agonist-resistant patients. Eur J Endocrinol. (2012) 166:779–86. 10.1530/EJE-11-1000 22301915

